# On-Chip Free-Flow Measurement Revealed Possible Depletion of Macrophages by Indigestible PM2.5 within a Few Hours by the Fastest Intervals of Serial Phagocytosis

**DOI:** 10.3390/mi14010206

**Published:** 2023-01-13

**Authors:** Dan Horonushi, Yuya Furumoto, Yoshiki Nakata, Toshiki Azuma, Amane Yoshida, Kenji Yasuda

**Affiliations:** 1 Department of Pure and Applied Physics, Graduate School of Advanced Science and Engineering, Waseda University, 3-4-1 Okubo, Shinjuku, Tokyo 169-8555, Japan; 2 Department of Physics, School of Advanced Science and Engineering, Waseda University, 3-4-1 Okubo, Shinjuku, Tokyo 169-8555, Japan

**Keywords:** PM2.5, microplastic, phagocytosis, macrophage, indigestible antigen, free-flow method, optical tweezers, microneedle method

## Abstract

To understand the influence of indigestible particles like particulate matter 2.5 (PM2.5) on macrophages, we examined the time course of the series phagocytosis of indigestible 2 μm polystyrene spheres (PS). Five kinds of antigens were used as samples for phagocytosis; Zymosan, non-coated 2 μm PS, bovine serum albumin (BSA)-coated PS (BSA-PS), IgG-coated PS (IgG-PS), and IgG-BSA-coated PS (IgG/BSA-PS). To keep the surrounding concentration of antigens against single macrophages constant, antigens flowed at a continuous rate of 0.55 μm/s within a culture dish as a free-flow measurement assay (on-chip free-flow method). The interval of series phagocytosis for IgG/BSA-PS was the shortest among five samples; it was six times faster than Zymosan in terms of engulfment frequency, and up to 50 particles were engulfed within two hours, maintaining constant intervals until reaching the maximum number. The rate of increase in the total number of phagocytozed IgG/BSA-PS over time was constant, at 1.5 particles/min, in series phagocytosis with a 33-cell population, indicating that the phagocytosis rate constant remained constant independent of the number of phagocytoses. Reaction model fitting of the results showed that IgG/BSA-PS had the highest efficiency in terms of the phagocytosis rate constant, 2.3 × $10^{-2}$ particles/min, whereas those of IgG-PS, BSA-PS, PS, and Zymosan were 1.4 × $10^{-2}$, 1.1 × $10^{-2}$, 4.2 × $10^{-3}$, and 3.6 × $10^{-3}$ particles/min, respectively. One-by-one feeding of IgG/BSA-PS with optical tweezers was examined to confirm the phagocytosis intervals, and we found that the intervals remained constant until several times before the maximum number of antigens for engulfment, also indicating no change in the phagocytosis rate constant regardless of the history of former phagocytosis and phagocytosis number. Simultaneous phagocytosis of two IgG-BSA-decorated microneedle engulfments also showed that the initiation and progress of two simultaneous engulfments on the two different places on a cell were independent and had the same elongation velocity. Therefore, each phagocytosis of indigestible antigens does not affect both in series or in simultaneous subsequent phagocytosis until reaching the maximum capacity of the phagocytosis number. The results suggest (1) no change in the phagocytosis rate constant regardless of the history of phagocytosis numbers and attachment timing and positions, and (2) IgG-BSA decoration of indigestible microparticles in blood accelerates their engulfment faster, resulting in a severe shortage of macrophages within the shortest time.

## 1. Introduction

Macrophages are some of the primary innate immune cells that engulf and digest pathogens [1], and are also responsible for antigen presentation to activate the acquired immune system and cytokine secretion to mobilize other immune cells to inflammatory sites [2]. Hence, the depletion of macrophages induces severe immunodeficiency.

Phagocytosis is initiated when macrophages come into contact with target substances and recognize them as antigens, either directly or through the binding of opsonized molecules on the target surface to cell surface receptors [3]. Macrophages have multiple types of receptors on the plasma membrane, including Toll-like receptors (TLRs), scavenger receptors that primarily recognize denatured lipoproteins, and Fc receptors that recognize the Fc portion immunoglobulin, allowing them to target a wide variety of antigens [4]. In receptor-mediated phagocytosis, the zipper mechanism, in which successive binding between receptors and antigen surface ligands leads to antigen envelopment by the cell membrane, was proposed by Griffin and his colleagues in the 1970s and has since been supported by a large body of experimental evidence [5,6,7]. That is, the modification state of the ligand on the antigen surface affects phagocytosis; for example, phagocytosis of polystyrene particles of 0.5 μm to 2 μm diameter by macrophages has been reported to increase the number of phagocytes as the antibody density on the particle surface increases [8].

Recently, it has been reported that particulate matter 2.5 (PM2.5), as an air pollutant or in the form of microplastics in water, reduces the viability and activity of macrophages and inhibits immune system function [9,10,11,12]. A study examining the phagocytic ability of alveolar macrophages in rats injected with PM2.5 into the trachea once a week for eight weeks found that macrophages exposed to higher concentrations of PM2.5 lost more phagocytic response-ability to the antigen [13]. It has also been suggested that PM2.5 exposure may lead to an excessive immune response by disturbing the polarity balance between M1 and M2 macrophages, which produce inflammatory and anti-inflammatory cytokines, respectively, in a direction that enhances M1 polarization [11]. Thus, the effects of PM2.5 on macrophage phagocytic function and polarity have been extensively studied and unveiled gradually recently [14,15]. However, these studies contrast macrophages before and after PM2.5 exposure (in other words, in equilibrium), and there have been few studies that observe macrophages in a non-equilibrium state during PM2.5 exposure in real-time and follow the process of changes in their properties.

This study aims to identify the mechanism of phagocytosis’s ability to decrease and deplete macrophages as a result of exposure to PM2.5. PM2.5 entering the body is expected to be efficiently taken up by macrophages by being coated by serum albumin and opsonized molecules such as complement and antibodies. In addition, since PM2.5 is an indigestible particle, it may accumulate in the body of macrophages without being degraded, affecting subsequent phagocytosis by macrophages. Therefore, tracking how the phagocytosis rate changes depending on whether the particles are opsonized and on the previous phagocytosis hysteresis of macrophages will clarify the mechanism of macrophage dysfunction and depletion due to PM2.5.

In this study, we investigated the dependence of the macrophage phagocytosis rate on the antigen surface coating and phagocytosis hysteresis based on serial phagocytosis by a macrophage population of four types of indigestible polystyrene spheres (PS) of 2 μm diameter with different surface coatings of IgG, bovine serum albumin (BSA), and Zymosan. The results show that the phagocytosis rate depended on the antigen surface structure and that the rate for PS coated with IgG or BSA was more than twice that of uncoated PS or Zymosan. However, in one-by-one feeding, in which PS coated with both IgG and BSA were phagocytozed one after another by single macrophages until any response disappeared, the phagocytosis rate remained constant even as the number of phagocytozed PS accumulated, and only after a few times just before the phagocytic limit did the rate significantly decrease. When single macrophages simultaneously phagocytozed two glass microneedles coated with IgG and BSA from opposite directions, the cell membrane elongated independently and at the same rate on each needle. These results suggest that when PM2.5 indigestible particles enter the body, they can saturate the phagocytosis of macrophages at once in the shortest time by binding with BSA and IgG, thereby depleting macrophages, the initiators of the acquired immune system.

## 2. Materials and Methods

### 2.1. Cells

We used a mouse-derived macrophage-like cell line (J774.2; Sigma-Aldrich, St.Louis, MO, USA) as the macrophage model for this experiment. We incubated the J774.2 cell line at 37 °C under 5% CO_2_ using Dulbecco’s modified Eagle’s medium (DMEM; Gibco Thermo Fisher Scientific, Waltham, MA, USA) with 10% heat-inactivated fetal bovine serum (FBS; Gibco Thermo Fisher Scientific, Waltham, MA, USA), 100 units/mL penicillin (Gibco Thermo Fisher Scientific, Waltham, MA, USA), and 100 μg/mL streptomycin (Gibco Thermo Fisher Scientific, Waltham, MA, USA). For passage of the cells, peeling off from the bottom of the culture flask (AGC TECHNO GLASS Co., Ltd., Shizuoka, Japan) was carried out not with a protease such as trypsin but with a cell scraper (BM Equipment Co., Ltd., Tokyo, Japan) because some protease can damage the receptors on the cell. Considering the possibility of change in characteristics depending on the number of passages, we used up to the fifth generation at the maximum.

### 2.2. Antigens

**Figure 1:**
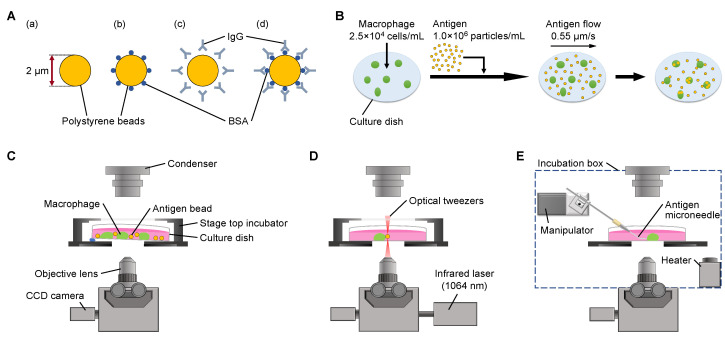
**Four kinds of antigen bead and measurement methods for antigen bead and microneedle phagocytosis**. (**A**). Schematic drawings of samples: (**a**) polystyrene (PS) bead, (**b**) BSA-PS bead, (**c**) IgG-PS bead, (**d**) IgG/BSA-PS bead. (**B**). Procedure of antigen bead phagocytosis experiment by the on-chip free-flow method. Antigen beads were flown in the dish with constant speed to keep the concentration of surrounding macrophages constant. (**C**). Experimental setup for free-flow method. Since macrophages adhered to the bottom of the dish, tilting the dish could generate a flow of antigen beads to the macrophages. (**D**). Experimental setup of bead feeding with optical tweezers. To feed antigen beads one-by-one to macrophages, the beads were trapped and transferred with optical tweezers. (**E**). Experimental setup for IgG/BSA-coated microneedle phagocytosis. The tip of the antigen microneedles was brought into contact with the macrophages by moving the motorized manipulator to which the antigen microneedle was attached.

We prepared five types of antigen models in this experiment: four types of PS with different decorations as shown in Figure 1A, Zymosan (FUJIFILM Wako Pure Chemical Corporation, Osaka, Japan) as the control. We also prepared IgG/BSA decorations for glass microneedles and IgG/BSA-glass microneedles for microneedle measurements.

We used 2 μm PS beads (Polysciences, Inc., Warrington, PA, USA) as the core of the four non-digestible types of antigen beads. Details of the four types of antigen beads were as follows: PS bead without decoration (Figure 1A(a)), PS beads coated with bovine serum albumin (BSA; Sigma-Aldrich, St.Louis, MO, USA) (BSA-PS bead) (Figure 1A(b)), PS beads coated with anti-BSA immunoglobulin G (IgG; Bethyl Laboratories, Inc., Montgomery, TX, USA) (IgG-PS bead) (Figure 1A(c)), and PS beads coated with BSA followed by IgG decoration (IgG/BSA-PS bead) (Figure 1A(d)).

PS beads were washed three times with phosphate-buffered saline (PBS; Takara Bio Inc., Shiga, Japan) (10,000× *g*, 5 min). BSA-PS beads were prepared by incubating washed PS beads in 10 mg/mL BSA/PBS solution for 24 h at room temperature and then rewashing three times with PBS. IgG-PS beads were prepared by incubating washed PS beads in 0.33 mg/mL IgG/PBS solution for one hour at room temperature and then rewashing three times with PBS. IgG/BSA-PS beads were prepared by incubating washed BSA-PS beads in 0.33 mg/mL IgG/PBS solution for one hour at room temperature and then rewashing three times with PBS. These antigen beads were stored in PBS to prevent drying and used at concentrations adjusted with PBS as appropriate. Zymosan was also used at concentrations adjusted with PBS.

IgG/BSA-glass microneedles were prepared by decorating the surface of glass microneedles made from 1 mm diameter glass capillary tubes (GD-1; Narishige, Tokyo, Japan) in the same way as IgG/BSA-PS beads. The 2–10 μm diameter glass microneedles were made from 1 mm diameter glass capillary tubes (GD-1; Narishige, Tokyo, Japan) with a micropipette puller (P-97; Sutter Instrument, Novato, CA, USA). The hollow of the micropipette end was closed by heating a microforge (MF-2; Narishige, Tokyo, Japan). Then, the microneedle was heated and bent by 30° at 200 μm from the tip (see Figure 1E). Microneedles were washed three times with PBS on a laboratory shaker (Wave-SI; TAITEC, Saitama, Japan) and then incubated in 10 mg/mL BSA/PBS solution for 24 h at room temperature. After the incubation, they were washed three times and incubated in 0.11 mg/mL IgG/PBS solution for one hour at room temperature. Finally, they were rewashed three times and stored in PBS to prevent drying.

For confirmation of the IgG decoration on the surface of IgG-PS beads, IgG/BSA-PS beads, and IgG/BSA-glass microneedles, a secondary antibody against IgG with Alexa Fluor 488 (Abcam, Cambridge, UK) was used and observed with a fluorescent invert optical microscope (IX71; Olympus, Tokyo, Japan).

### 2.3. On-Chip Free-Flow Method for Serial Phagocytosis Rate Constant Evaluation

The schematic design and procedure of the on-chip free-flow method are shown in Figure 1B,C. First, $5.0\times 10^{4}$ cells were placed in the 35 mm cell-culture dish (AGC TECHNO GLASS Co., Ltd., Shizuoka, Japan) with 2 mL DMEM and incubated for 3 h at 37 °C under 5% CO_2_ to allow them to adhere to the bottom of the dish. Then, $2.0\times 10^{6}$ antigen beads were added to the dish carefully, and it was placed on the inverted microscope (IX71; 10× obj. lens; Olympus, Tokyo, Japan) stage with a slight tilt (see Figure 1C) to form 0.55 μm/s of antigen flow before phagocytosis of the macrophages had begun. Since macrophages adhered to the bottom of the dish, tilting the cultivation dish generated a flow of antigen beads to the macrophages.

The observation was started as soon as the dish was set on the stage, and the concentration of antigen beads and the percentage of phagocytic cells in the observed area at each time were measured. During the experiment, the dish was placed in an appropriate environment for cell activity (37 °C, 5% CO_2_) by a stage-top incubator (Tokai Hit., Co, Ltd., Shizuoka, Japan). The microscope was equipped with a charge-coupled device camera (FX630, CCD camera; Olympus, Tokyo, Japan) to take micrographs at each time.

### 2.4. Optical Trapping of IgG-Coated Polystyrene Spheres for Serial Phagocytosis Measurement

We used an optical tweezer system (LMS-44021; SIGMA KOKI Co., Ltd., Tokyo, Japan) built into the inverted microscope (IX71; 100× obj. lens; Olympus, Tokyo, Japan) to evaluate series phagocytosis of single macrophages as shown in Figure 1D. First, $5.0\times 10^{4}$ cells were placed in the 35 mm cell-culture dish with 2 mL DMEM and incubated for 3 h at 37 °C under 5% CO_2_. Then, a small number of IgG/BSA-PS beads were added to the dish carefully. Next, the dish was set on the inverted microscope stage, and IgG/BSA-PS beads were picked up and brought to the macrophage one-by-one after each phagocytosis was finished under 37 °C, 5% CO_2_ conditions. The phagocytosis process was observed with a ×100 objective lens and recorded with a CCD camera every 5 s interval for time-lapse recording.

### 2.5. IgG-Coated Microneedle Phagocytosis for Cell Membrane Progress Speed Evaluation

As the complementary experiment to the above experiments, IgG/BSA-glass microneedles were also used for simultaneous phagocytosis experiments in the setup, as shown in Figure 1E. First, $7.5\times 10^{4}$ cells were placed in the 60 mm cell-culture dish (BM Equipment Co., Ltd., Tokyo, Japan) with 5 mL DMEM and incubated for 3 h at 37 °C under 5% CO_2_. The incubated cultivation dish was placed on the inverted microscope (IX70; 40× objective lens; Olympus, Tokyo, Japan) stage shown in Figure 1E. Then, IgG/BSA-glass microneedles were attached to a macrophage in the dish using a motorized manipulator (Micromanipulator 5171; Eppendorf, Hamburg, Germany). The phagocytosis process was recorded with a CCD camera (1500M-GE, CCD camera; Thorlabs, Newton, NJ, USA) every 5 s for time-lapse recording.

## 3. Results and Discussion

### 3.1. Evaluation of Free-Flow Method for Stationary Supply of Antigens for Phagocytosis Rate Constant Measurement

In the conventional dish cultivation method, in which macrophages and antigen beads were co-cultivated, the concentration of antigen beads around the macrophages decreased during their phagocytosis. As macrophages might become unable to phagocytose before they reach their actual limit of phagocytosis numbers due to the depletion of the surrounding beads, we might underestimate their phagocytic capacity and phagocytosis rate constant.

To overcome this problem, we developed a simple method to maintain the concentration of antigen beads constantly surrounding the macrophages during the experiments by flowing the antigen beads with a constant slow speed as the on-chip free-flow method (Figure 1B). Using this method, we were able to keep the concentration of antigens around each macrophage constant to determine the saturated maximum limit of phagocytosis and their efficiencies.

**Figure 2:**
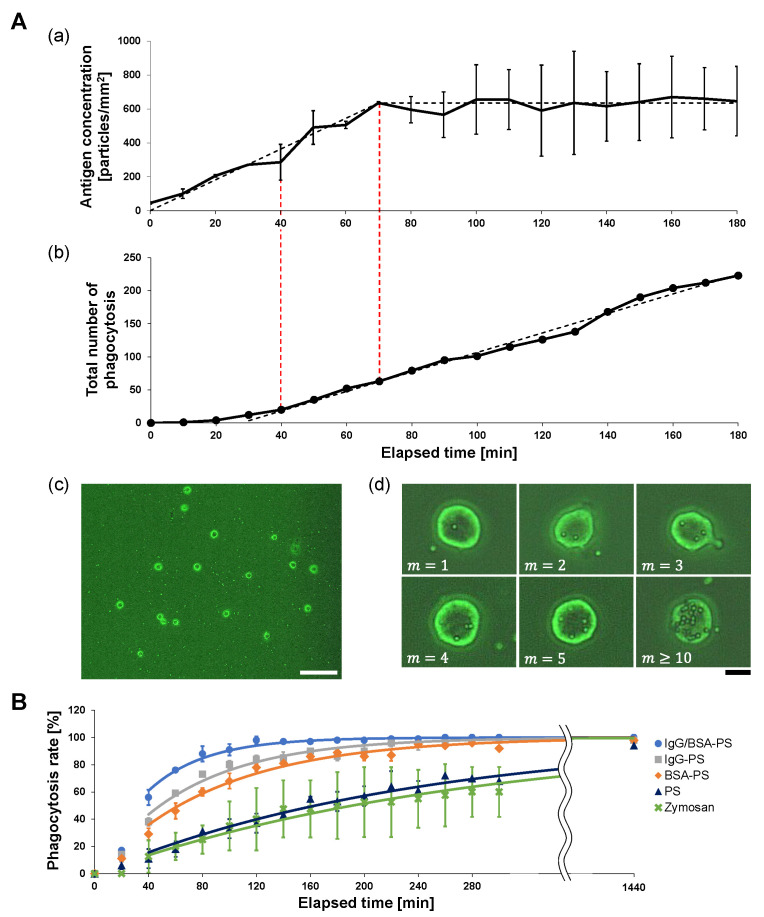
**Time-course change in antigen concentration and total number of phagocytozed macrophages in the on-chip free-flow method.** (**A**). Time-course change in antigen bead concentration and the total number of antigen beads phagocytozed by macrophages. (**a**) Time-course change in flowing antigen concentration at the observation area. The concentration of flowing antigens became stable 70 min after the flow started. The black dashed line is the model line when the antigen concentration is assumed to increase at a constant rate during the initial 70 min toward the bead concentration after 70 min (635 particles/mm^2^) and then remain constant. (**b**) Total number increase of antigens phagocytozed by 33 macrophages. The black dashed line is the fitting line using the least-squares method for the plot after 40 min when the rate of increase in the number of phagocytozed beads became constant. The slope of the dashed line is 1.5/min. (**c**) Micrograph of the entire observation area at 70 min when the antigen concentration became constant. Bar: 100 μm. (**d**) Micrograph of macrophages phagocytosing antigen beads. The *m* in the lower-left of each picture represents the engulfed antigen number. Bar: 10 μm. (**B**). Sample surface decoration dependence of phagocytic efficiency. The phagocytic efficiency of five types of antigens was compared: IgG/BSA-PS (light blue dots, error bars and line, n = 100 cells), IgG-PS (gray, n = 100), BSA-PS (orange, n = 100), PS (dark blue, n = 100), and Zymosan (green, n = 40). The dots and error bars represent the mean and standard deviation of the experimental values, respectively, and the lines are the fitting curves according to Equation (6). The phagocytosis ratio at each time represents the percentage of macrophages that phagocytozed at least one antigen relative to the total number of macrophages. Since Zymosan is a digestible biological sample, data for 1440 min could not be obtained because it is not possible to confirm by direct observation whether or not the macrophages after an extended period are cells after phagocytosis.

First, we evaluated the ability of the on-chip free-flow method to supply antigens constantly to macrophages. Figure 2A(a) shows an example of the time-course change of the 2 μm IgG/BSA-PS bead concentration on the bottom of the dish measured every 10 min from the start of the experiment. Under the 0.55 μm/s flow of antigens, the concentration increased linearly with a 9.1 particles/mm^2^ · min ratio to the stable concentration of 635 particles/mm^2^ during the initial 70 min. The dashed line is the reference line assuming that the antigen concentration increased with 8.9 particles/mm^2^ · min during the initial 70 min toward the stable concentration (623 particles/mm^2^) and then remained constant.

Figure 2A(b) shows the time-course increase in the total number of antigens engulfed by macrophages with 52 cells/mm^2^ on the dish (n = 33). The total number of engulfed antigen beads increased and finally turned into a linear increase with 0.35 particles/cell/min or 1.8 particles/mm^2^/min or 40 min after measurement started, which was earlier than the time of the antigen concentration constant. This engulfment efficiency was maintained until all macrophages reached their limit of engulfment. The graph’s dashed line is the fitting reference line for the constant rate increase in phagocytozed beads.

Compared with Figure 2A(a), the supply of antigens with a 0.55 μm/s flow was enough to maintain the surrounding antigen constant at 623 particles/mm^2^ and was also beyond the phagocytosis rate constant 0.14 particles/cell/min of 52 cells/mm^2^ macrophages. Hence, we can regard that the on-chip free-flow system can supply enough antigens for macrophages to phagocytoze with the highest phagocytosis rate constants with the saturated number of surrounding antigens during this experiment.

### 3.2. Time-Course Phagocytosis Number Measurement of Antigens with On-Chip Free-Flow Method: Sample Surface Decoration Dependence of Phagocytosis Efficiency

Next, we observed macrophage phagocytosis in the on-chip free-flow method for each of the four kinds of antigen bead and Zymosan to evaluate the difference in phagocytosis efficiency depending on the surface decoration of antigens. The bottom of the dish was observed at the fixed area every 20 min after antigen flow was started, and the percentage of phagocytic cells, macrophages that phagocytozed at least one antigen, to the total number of cells in the observation area was measured. We named this rate the “phagocytosis rate” and used it as an index of the phagocytosis efficiency of the macrophages. By comparing the phagocytosis rate and its time change among antigens, we were able to quantitatively evaluate the phagocytozed efficiency of each antigen.

Figure 2B shows the time-course increase of the phagocytozed macrophage number for five types of antigens: IgG/BSA-PS (blue line, n = 100), IgG-PS (gray, n = 100), BSA-PS (red, n = 100), PS (yellow, n = 100), and Zymosan (green, n = 40). The phagocytic efficiency of those five types of antigens for macrophages was estimated as the ratio at each time representing the percentage of macrophages that phagocytozed at least one antigen relative to the total number of macrophages in the observation region. The results show that the increase in the phagocytozed macrophage number was larger in the order of IgG/BSA-PS bead > IgG-PS bead > BSA-PS bead > PS bead > Zymosan, indicating that the phagocytosis rate constant of the four types of PS beads for macrophages should be different depending on the surface decoration of antigens. The fastest was for the IgG/BSA-PS beads because these rate constants should be the same as the increase in the phagocytozed macrophage numbers. As the four types of indigestible PS beads remained in macrophages after phagocytosis, the total number of phagocytozed macrophages reached 100% after 1440 min cultivation, regardless of the decoration on the PS surfaces. In contrast, since Zymosan is a digestible biological sample, the observed number of phagocytozed macrophages reached 60% and was saturated because Zymosan phagocytozed in the cell was digested.

### 3.3. Rate Constant Estimation of Phagocytosis in Five Types of Antigens

To quantitatively estimate the time-course rate of the first phagocytosis in five types of antigens observed in Figure 2B, we assumed a simple chemical-reaction-like model in phagocytosis and calculated the phagocytosis rate constant of macrophages to be able to compare the differences between the five types of antigens.

In the model, the cell concentration and the surrounding antigen concentration determined the reaction rate constant between the cells and each antigen. The number ratio of macrophages engulfed in at least one antigen $N_{m\geq 1}\left(t\right)$ against the total number of intact macrophages in the observation area *N* at time *t* is described as,

(1)Nm≥1(t)N=Nm≥1(t−1)N+kmaxNm=0(t−1)N(2)=Nm≥1(t−1)N+kmax(1−Nm≥1(t−1)N)(3)=kmax−(1−k·c)Nm≥1(t−1)N(4)=1−(1−kmax)t

where *m* is the number of engulfed antigens in a macrophage, $k_{max}$ is the maximum phagocytosis rate constant of macrophages surrounded with a saturated antigen concentration, and *N* is the total number of macrophages. In this model, we applied the concentration condition of the surrounding antigens being constant as *c* because of the continuous supply of antigens. We also supposed that the phagocytosis rate constant $k_{max}$ is independent of the engulfed antigen number *m*. As we set the initial conditions $N_{m\geq 1}\left(0\right)=0$, the time course of the engulfed macrophage number is described in Equation (4). We can also replot Equation (2) in the differential equation. When we use $x\left(t\right)=\frac{N_{m\geq 1}\left(t\right)}{N}$, the equation is simplified in typical rate equation formula as,

(5) $$\begin{array}{cc} \frac{dx(t)}{dt} & =k_{max}\left(1-x\left(t\right)\right). \end{array}$$

Hence, the increase curve of the phagocytozed macrophage number ratio is exponentially described in,

(6) $$\begin{array}{cc} x(t) & =1-e^{-k_{max}t}. \end{array}$$

We used $k_{max}$ of the five types of antigens as a quantitative index of phagocytosis efficiency. When we used the above Equation (6) for the fitting with the acquired data in Figure 2B under the constant increase time region from 40 min after measurement started, the acquired maximum phagocytosis rate constant for each type of antigen is summarized in Table 1. As shown in this table, the efficiency of phagocytosis in IgG/BSA-PS beads had a maximum speed of engulfment and was about two times faster than IgG-PS or BSA-PS and also ten times larger than non-coated PS and Zymosan. This means that macrophages engulfed indigestible IgG/BSA-PS beads with the highest efficiency of phagocytosis rather than engulfing Zymosan if the rate constant of those particles was independent of the engulfed number of indigestible antigens in each macrophage, as we assumed for the above estimation.

It should be noted that the possibility of underestimation of the rate constant of Zymosan because of the efficiency of engulfment was based on the number counting of Zymosan particles in macrophages. Hence, the digestion of Zymosan reduced the estimation of total phagocytosis intervals.

### 3.4. Phagocytosis Number Dependence of Phagocytosis Efficiency: One-By-One Feeding Evaluation

In the above phagocytosis rate constant estimation, we supposed that the ability and efficiency of phagocytosis were independent of the phagocytosis number. To confirm this hypothesis, we examined their difference during serial one-by-one feeding of antigens for single macrophages using optical tweezers, as shown in Figure 1D. For this experiment, IgG/BSA-PS beads were chosen as antigen beads because phagocytosis experiments using the free-flow method described above showed that IgG/BSA-PS beads are phagocytozed by macrophages most efficiently among the four kinds of antigen beads.

**Figure 3:**
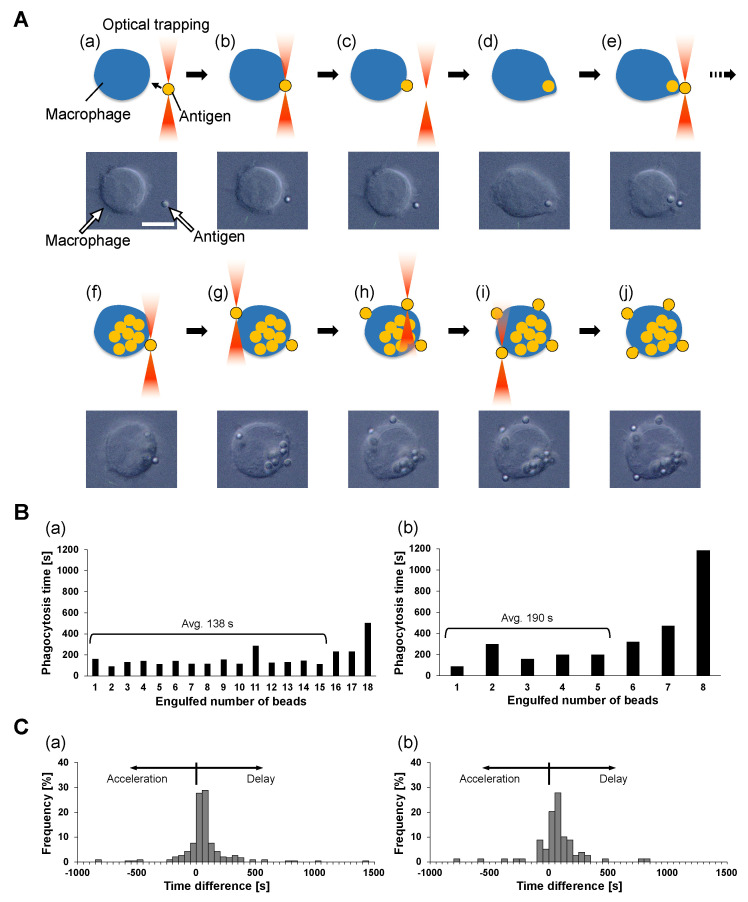
**Evaluation of phagocytic antigen number dependence of phagocytosis time after feeding with optical tweezers.** (**A**). Schematic drawing and micrographs of one-by-one phagocytosis process. (**a**) Transport of first antigen IgG/BSA-PS bead with optical tweezers. (**b**) Contact of first antigen bead with cell. (**c**) Removal of optical trapping after antigen bead adhesion. (**d**) Completion of phagocytosis for first bead. (**e**) Contact of second antigen bead. (**f**) Contact of antigen bead after the macrophage reached its maximum limit of phagocytosis. (**g**–**i**) Contact of additional beads on the other areas of the cell. (**j**) Final state. Bar: 10 μm. (**B**). Typical example of phagocytosis number dependence of phagocytosis time in 2 μm (**a**) and 4.5 μm (**b**) IgG/BSA-PS bead phagocytosis, respectively. (**C**). Histogram of difference in phagocytosis time in adjacent phagocytosis in 2 μm ((**a**), n = 235 phagocytosis) and 4.5 μm ((**b**), n = 79) IgG/BSA-PS bead phagocytosis, respectively.

As shown in Figure 3A, the one-by-one feeding process of antigen beads was operated as follows. First, an antigen bead, the first bead, was captured and brought into contact with the cell surface with optical tweezers (a and b). After confirming the adhesion of the first bead to the cell, the bead was released from the optical tweezers (c) and internalized into the cell (d). Similarly, the second bead was captured and brought to the same place on the cell surface (e). There was a short lag time for the phagocytosis interval between the completion of the first bead phagocytosis and the contact of the second bead. In the same way, contact and internalization were carried out for the third and subsequent beads until the cell reached its maximum limit of phagocytosis. We confirmed the mineralization of the last antigen bead by the following procedure. First, an antigen bead was attached to the same point on the cell at which the previous beads were brought into contact (f), and we confirmed that this bead was not phagocytozed for more than 30 min. Then, an additional bead was brought into contact with an opposite point from the previous one (g), and no response for 30 min was confirmed again. Finally, we added two more antigens to the other two points between these two beads similarly and checked for no responses for 30 min (h–j).

These one-by-one feeding results also indicate the interesting characteristics of engulfment. As shown in the above results, once the macrophage reaches the maximum limit of engulfment of non-digestive antigens on the part of the cell surface, the macrophage does not start the next engulfment in the place of the last phagocytosis or on any other part of the cell surface. This means the regulation of the maximum limit of phagocytosis is not caused by the local membrane capacity limit or local receptor concentration but is caused by the information of the whole cell volume increase.

Figure 3B shows typical examples of the phagocytosis number dependence of the time required for the completion of phagocytozing of 2 μm and 4.5 μm beads during the series one-by-one attachment. During the interval of phagocytosis of each bead by macrophages, the time from adhesion of the beads to the cell membrane (Figure 3A(c)) to completion of bead uptake into the cell (Figure 3A(d)) was measured and defined as the phagocytosis time. The two bar graphs show that the phagocytosis time was similar with fluctuations independent of the phagocytosis number except for the final few phagocytoses, which took longer than the earlier ones.

To confirm this consistency of phagocytosis time, we replotted the difference in phagocytosis times between adjacent phagocytoses as the histograms in Figure 3C. These histograms show that phagocytosis times tend to be slightly delayed, on average, 17.6 s and 18.7 s in (a) and (b), respectively. However, in (a), 68.1% of the total serial phagocytosis shows a time difference of 77.5 s (half of the median of the phagocytosis time) or less, and in (b), 63.3% of the total cases show a phagocytosis time difference of 102.5 s or less.

Hence, we concluded that the required time for phagocytosis is independent of their number in the series phagocytosis. One-by-one feeding results show that the phagocytosis rate of macrophages remains constant, independent of their previous phagocytosis history. Therefore, the assumption in the previous section suggests that the phagocytosis rate constant $k_{max}$ of indigestible polystyrene beads is independent of the engulfed antigen number *m* as long as the macrophages do not reach their phagocytosis number limit.

### 3.5. Evaluation of Independence of Local Engulfments in Space: Simultaneous Two-Microneedle Phagocytosis Assay

As shown above, each phagocytosis was independent of their number during the series of one-by-one phagocytoses. Then, we might ask, what about the difference in simultaneous phagocytosis? We applied the IgG-coated two-microneedle phagocytosis experiment to examine the difference in two simultaneous phagocytoses on a single macrophage.

**Figure 4:**
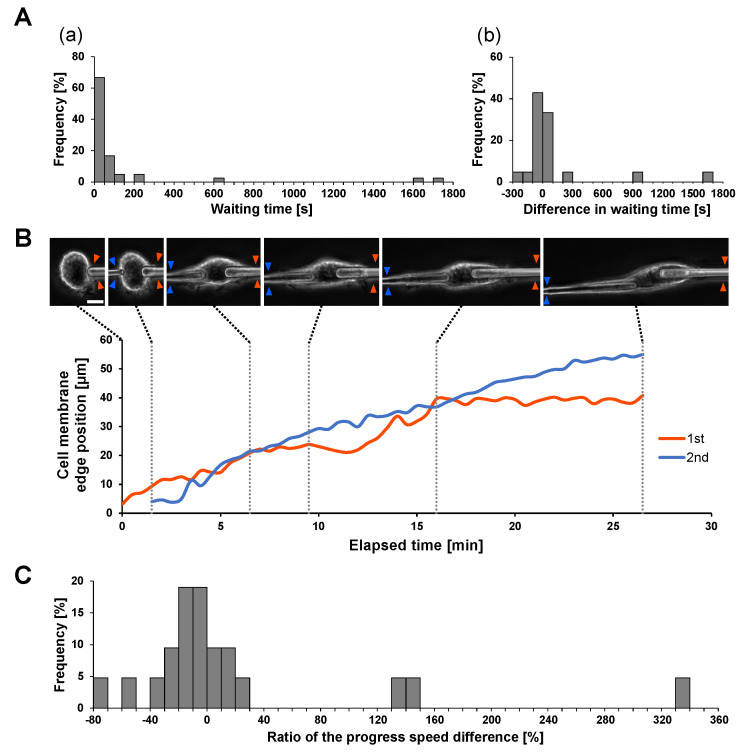
**Waiting time from antigen contact to the start of phagocytosis and progress speed in IgG/BSA-coated microneedle phagocytosis.** (**A**). Evaluation of waiting time from antigen microneedle contact to the start of phagocytosis in two-microneedle phagocytosis (n = 21 samples). (**a**) Histogram of waiting time. The time from microneedle contact to the start of phagocytosis was measured for each of the two needles. (**b**) Difference in waiting time in the first and second needles. (**B**). Typical example of cell membrane progression on the first (red) and second (blue) microneedles in two-microneedle phagocytosis. The edges of the cell membrane on the first and second microneedles are indicated by red and blue arrowheads in the micrographs. Bar: 10 μm. (**C**). Evaluation of the difference in the progress speed of the cell membrane on the first and second microneedles. The progress speed was measured for each of the two microneedles, and the ratio of these differences to the progress speed on the first needle, i.e., 100 × ((2nd) − (1st))/(1st), was calculated.

Figure 4A shows the required time for initiation of engulfment of two microneedles (A) and their differences (b) in 21 experiments. As shown in the bar graphs, the local responses of macrophages in different places showed no significant differences. Then, we compared the difference in internalization efficiency in simultaneous engulfment by comparing the speed of the cell membrane extension on the IgG-coated microneedle. Figure 4B shows an example of two microneedle experiments. As shown in the micrographs and the graph, the velocities of cell membrane extensions on the two microneedles were similar, at 0.0390 μm/s (1st) and 0.0338 μm/s (2nd), respectively. This similarity is also supported in Figure 4C. These results also indicate that the internalization efficiency in simultaneous phagocytosis is similar and the results are independent of each other.

Therefore, the estimation of the phagocytosis rate constant $k_{max}$ in the on-chip free flow does not need to consider the engulfment intervals or the possibility of simultaneous occurrence.

### 3.6. Possible Depletion of Macrophages by PM2.5

In this study, we demonstrated that indigestible 2 μm polystyrene spheres with surface decorations of blood components such as BSA and IgG showed a much higher phagocytosis rate constant than natural digestible antigens like Zymosan. Significantly, the beads decorated with IgG and BSA maintained a stable high phagocytosis rate constant until the macrophages almost reached their saturation, which was more than six times the efficiency of non-coated polystyrene spheres and Zymosan.

Our results in one-by-one feeding also showed the deactivation of macrophages when they engulfed nondigestible IgG/BSA-PS beads to their maximum number limit.

These two pieces of evidence suggest the possibility of depletion of macrophages when we apply sufficient nondigestible PM2.5 into the vascular system. As the macrophages engulfed IgG/BSA-coated nondigestible microparticles with high frequency, a shortage of active macrophages might occur in our immune system.

When foreign matter enters the body, it does not remain at a static site but moves through bodily fluids, such as lymph and blood circulations. According to the affinity of those microparticle surfaces to the blood components, a reasonable amount of particulate matter should be opsonized to become antigens. Therefore, macrophages should engulf those microparticles, and most of the saturated macrophages proceed to apoptosis.

Many reports have shown the effect of PM2.5 on the functions of macrophages. For example, macrophages exposed to PM2.5 have impaired viability [9,10,16,17], ability to respond to antigens or phagocytic capacity [12,16,18], production of cytokines and nitric oxide, which induce inflammation and increase the bactericidal activity of immune cells [12], and ability to activate lymphocytes [16]. These impaired functions can cause a significant reduction in host defense against infection since they result in impaired mobilization and activation of other immune cells, as well as clearance of antigens by the macrophages themselves. This reduction of host defenses can continue for as long as a week after exposure to PM2.5 [16].

All the above evidence is consistent with our results. Because PM2.5 particles are indigestible particles, macrophages saturated with phagocytosis by PM2.5 are unable to digest the particles they have taken in and regain their phagocytic capacity, which eventually leads to apoptosis, resulting in the impairment of cell viability and phagocytic function. In addition, since antigen presentation, which is the process of lymphocyte activation, and secretion of inflammation-inducing substances are both based on antigen information phagocytozed by macrophages, if the phagocytic function is impaired, they will be impaired accordingly. In the present study we showed that phagocytic saturation of macrophages by PM2.5 is completed in the shortest time when PM2.5 is modified by BSA or IgG. In other words, macrophages that respond to large amounts of PM2.5 at certain local sites in the body, for example, in the alveoli, can be depleted before calling for the support of other immune cells. The time required for the host to recognize this local depletion of macrophages and to clear depleted cells and replenish new macrophages will be more than that for normal inflammation with pathogens as antigens, and thereby the host’s defenses will stagnate for some time; for example, one week shown by previous studies.

However, PM2.5 can also cause diseases and disorders due to immune overactivity. For example, it has been reported that PM2.5 can cause an increase in M1 macrophages, which leads to immune hyperactivity [11], and exacerbates lipid accumulation in macrophage foam cells, which causes atherosclerosis [17]. In addition, adverse effects not only on macrophages but also on the immune system as a whole (immunotoxicity) and damage to the cardiovascular system have been pointed out [19,20]. For example, a study measuring CD4(+)T cell counts and HIV viral load in the blood of HIV/AIDS patients exposed to PM2.5 showed that CD4(+)T cells decreased and HIV viral load increased as the concentration of PM2.5 in the air increased [19]. Although this study focused on macrophage dysfunction and inactivity, the application of the free-flow method devised in this study is expected to elucidate the occurrence mechanisms of these reported disorders of polarity, an effector of mitochondrial damage on phagocytosis, and the hyperactivity of macrophages [21].

## 4. Conclusions

The on-chip free-flow measurement and complementary one-by-one feeding method, and simultaneous two-microneedle phagocytosis measurement, revealed that the hysteresis-free high phagocytosis rate constant of IgG-BSA coated indigestible 2 μm polystyrene particles induces a serious shortage of macrophages in body, which can damage the immune host defense system.

## Figures and Tables

**Table 1:** Phagocytosis rate constants in five types of antigens.

Antigen	Phagocytosis Rate Constant	Ratio
	(1/min)	
IgG/BSA-PS	$2.3\times 10^{-2}$	1
IgG-PS	$1.4\times 10^{-2}$	0.61
BSA-PS	$1.1\times 10^{-2}$	0.47
PS	$4.2\times 10^{-3}$	0.18
Zymosan	$3.6\times 10^{-3}$	0.15

## Data Availability

Not applicable.

## References

[1] Gordon S. (2016). Phagocytosis: An Immunobiologic Process. Immunity.

[2] Rosales C., Uribe-Querol E. (2017). Phagocytosis: A Fundamental Process in Immunity. BioMed Res. Int..

[3] Tollis S., Dart A.E., Tzircotis G., Endres R.G. (2010). The zipper mechanism in phagocytosis: Energetic requirements and variability in phagocytic cup shape. BMC Syst. Biol..

[4] Chandler D.B., Kennedy J.I., Fulmer J.D. (1986). Studies of membrane receptors, phagocytosis, and morphology of subpopulations of rat lung interstitial macrophages. Am. Rev. Respir. Dis..

[5] Griffin F.M., Griffin J.A., Leider J.E., Silverstein S.C. (1975). Studies on the mechanism of phagocytosis. I. Requirements for circumferential attachment of particle bound ligands to specific receptors on the macrophage plasma membrane. J. Exp. Med..

[6] Griffin F.M.J., Griffin J.A., Silverstein S.C. (1976). Studies on the mechanism of phagocytosis. II. The interaction of macrophages with anti-immunoglobulin IgG-coated bone marrow-derived lymphocytes. J. Exp. Med..

[7] Swanson J.A., Baer S.C. (1995). Phagocytosis by zippers and triggers. Trends Cell Biol..

[8] Pacheco P., White D., Sulchek T. (2013). Effects of Microparticle Size and Fc Density on Macrophage Phagocytosis. PLoS ONE.

[9] Xing Y.F., Xu Y.H., Shi M.H., Lian Y.X. (2016). The impact of PM2.5 on the human respiratory system. J. Thorac. Dis..

[10] Meng Z., Zhang Q. (2007). Damage effects of dust storm PM2.5 on DNA in alveolar macrophages and lung cells of rats. Food Chem. Toxicol..

[11] Zhao Q., Chen H., Yang T., Rui W., Liu F., Zhang F., Zhao Y., Ding W. (2016). Direct effects of airborne PM2.5 exposure on macrophage polarizations. Biochim. Biophys. Acta Biomembr..

[12] Chen Y.W., Huang M.Z., Chen C.L., Kuo C.Y., Yang C.Y., Chiang-Ni C., Chen Y.Y.M., Hsieh C.M., Wu H.Y., Kuo M.L. (2020). PM2.5 impairs macrophage functions to exacerbate pneumococcus-induced pulmonary pathogenesis. Part. Fibre Toxicol..

[13] Huang N.H., Wang Q., Xu D.Q. (2008). Immunological Effect of PM2.5 on Cytokine Production in Female Wistar Rats. Biomed. Environ. Sci..

[14] Sharma J., Parsai K., Raghuwanshi P., Ali S.A., Tiwari V., Bhargava A., Mishra P.K. (2021). Emerging role of mitochondria in airborne particulate matter-induced immunotoxicity. Environ. Pollut..

[15] Hetzel M., Ackermann M., Lachmann N. (2021). Beyond “Big Eaters”: The Versatile Role of Alveolar Macrophages in Health and Disease. Int. J. Mol. Sci..

[16] Migliaccio C.T., Kobos E., King Q.O., Porter V., Jessop F., Ward T. (2013). Adverse effects of wood smoke PM2.5 exposure on macrophage functions. Inhal. Toxicol..

[17] Liu J., Liang S., Du Z., Zhang J., Sun B., Zhao T., Yang X., Shi Y., Duan J., Sun Z. (2019). PM2.5 aggravates the lipid accumulation, mitochondrial damage and apoptosis in macrophage foam cells. Environ. Pollut..

[18] Zhou H., Lobzik L. (2007). Effect of concentrated ambient particles on macrophage phagocytosis and killing of Streptococcus pneumoniae. Am. J. Respir. Cell Mol. Biol..

[19] Liang W., Wang X., Xie N., Yan H., Ma H., Liu M., Kong W., Zhu Z., Bai W., Xiang H. (2023). Short-term associations of PM2.5 and PM2.5 constituents with immune biomarkers: A panel study in people living with HIV/AIDS. Environ. Pollut..

[20] Feng S., Huang F., Zhang Y., Feng Y., Zhang Y., Cao Y., Wang X. (2023). The pathophysiological and molecular mechanisms of atmospheric PM2.5 affecting cardiovascular health: A review. Ecotoxicol. Environ. Saf..

[21] Chodari L., Aytemir M.D., Vahedi P., Alipour M., Vahed S.Z., Khatibi S.M.H., Ahmadian E., Ardalan M., Eftekhari A. (2021). Targeting mitochondrial biogenesis with polyphenol compounds. Oxidative Med. Cell. Longev..

